# Fine mapping and identification of candidate genes for a QTL affecting *Meloidogyne incognita* reproduction in Upland cotton

**DOI:** 10.1186/s12864-016-2954-1

**Published:** 2016-08-08

**Authors:** Pawan Kumar, Yajun He, Rippy Singh, Richard F. Davis, Hui Guo, Andrew H. Paterson, Daniel G. Peterson, Xinlian Shen, Robert L. Nichols, Peng W. Chee

**Affiliations:** 1 Cotton Molecular Breeding Laboratory, University of Georgia, Tifton, GA 31793 USA; 2 USDA-ARS, Crop Protection and Management Research Unit, Tifton, GA 31793 USA; 3 Plant Genome Mapping Laboratory, University of Georgia, Athens, GA 30602 USA; 4 Institute for Genomics, Biocomputing & Biotechnology, Mississippi State University, Mississippi State, MS 39762 USA; 5 Cotton Incorporated, 6399 Weston Parkway, Cary, NC 27513 USA; 6 Present Address: Biotechnology Institute, Jiangsu Academy of Agricultural Sciences, Nanjing, 210014 China; 7 Present Address: College of Agronomy and Biotechnology, Southwest University, Chongqing, 400716 China

**Keywords:** Cotton, Root Knot Nematodes, RKN, QTLs, SNP, Candidate genes

## Abstract

**Background:**

The southern root-knot nematode (*Meloidogyne incognita*; RKN) is one of the most important economic pests of Upland cotton (*Gossypium hirsutum* L.). Host plant resistance, the ability of a plant to suppress nematode reproduction, is the most economical, practical, and environmentally sound method to provide protection against this subterranean pest. The resistant line Auburn 623RNR and a number of elite breeding lines derived from it remain the most important source of root-knot nematode (RKN) resistance. Prior genetic analysis has identified two epistatically interacting RKN resistance QTLs, *qMi-C11* and *qMi-C14,* affecting gall formation and RKN reproduction, respectively.

**Results:**

We developed a genetic population segregating only for the *qMi-C14* locus and evaluated the genetic effects of this QTL on RKN resistance in the absence of the *qMi-C11* locus. The *qMi-C14* locus had a LOD score of 12 and accounted for 24.5 % of total phenotypic variation for egg production. In addition to not being significantly associated with gall formation, this locus had a lower main effect on RKN reproduction than found in our previous study, which lends further support to evidence of epistasis with *qMi-C11* in imparting RKN resistance in the Auburn 623RNR source. The locus *qMi-C14* was fine-mapped with the addition of 16 newly developed markers. By using the reference genome sequence of *G. raimondii,* we identified 20 candidate genes encoding disease resistance protein homologs in the newly defined 2.3 Mb region flanked by two SSR markers. Resequencing of an RKN resistant and susceptible *G. hirsutum* germplasm revealed non-synonymous mutations in only four of the coding regions of candidate genes, and these four genes are consequently of high interest.

**Conclusions:**

Our mapping results validated the effects of the *qMi-C14* resistance locus, delimiting the QTL to a smaller region, and identified tightly linked SSR markers to improve the efficiency of marker-assisted selection. The candidate genes identified warrant functional studies that will help in identifying and characterizing the actual *qMi-C14* defense gene(s) against root-knot nematodes.

**Electronic supplementary material:**

The online version of this article (doi:10.1186/s12864-016-2954-1) contains supplementary material, which is available to authorized users.

## Background

Plant-parasitic nematodes are important pests in agriculture and are found across a broad range of climatic conditions. The genus *Meloidogyne* includes more than 70 species; four species – *M. incognita, M. arenaria, M. javanica* and *M. hapla* – account for approximately 95 % of the total crop area infested by this genus [1]. The Southern root-knot nematode (*Meloidogyne incognita*, RKN) is the most important parasitic nematode of Upland cotton inflicting economic losses through direct damage to the plant root system and indirectly through increasing severity of other root diseases such as Fusarium Wilt caused by *Fusarium oxysporum* f. sp. *vasinfectum*. Yield loss due to RKN has dramatically increased in the U.S. from 1 % in 1987 to 5.5 % in 2006, resulting in losses of more than 300 million pounds (4.4 %) of cotton valued over $235 million (Cotton Disease Loss Estimate Committee Report 2012). Recommended management practices to control RKN include crop rotation and nematicide application. The broad host-range of RKN leaves cotton growers with few profitable options to adopt crop rotation as a means of nematode management [2]. Nematicides applied at planting fail to provide season long protection; moreover, the future availability of most widely used cotton nematicide, aldicarb, is uncertain.

Host plant resistance can be an efficient RKN management tool in cotton production. Resistant varieties can offer good to excellent pest control efficacy and are also environmentally sound alternatives. Both cultivated and wild relatives of Upland cotton have been screened to identify germplasm displaying high levels of RKN resistance [3], and a few resistance sources were identified. Currently, the Auburn 623 RNR germplasm line is the most important source of RKN resistance because it displays near immunity to RKN infection. The resistance in Auburn 623 RNR is believed to have originated from transgressive segregation in a cross between two moderately resistant parents, Clevewilt 6 and Wild Mexican Jack Jones [4, 5]. Resistance in Auburn 623 RNR was transferred to several adapted cultivars through backcrossing, resulting in the release of M-lines with improved agronomic qualities while retaining near-immunity to RKN [4].

Quantitative trait locus (QTL) mapping studies have identified regions on the long arm of Chromosome 11 and the short arm of Chromosome 14 that confer RKN resistance in Auburn 623 RNR derived M-lines [6–8]. Origin of the resistance loci was traced to the two moderately resistant parents of Auburn 623 RNR, with the *qMi-C11* locus on Chromosome 11 inherited from Clevewilt 6 [8] and the *qMi-C14* locus on Chromosome 14 inherited from Wild Mexican Jack Jones [7, 8]. The *qMi-C11* locus was recently fine mapped to a 3.6 cM interval [6], however the QTL region near *qMi-C14* remains sparsely mapped [8, 9].

Recent studies suggested that the *qMi-C11* locus predominanly affects root gall suppression whereas the *qMi-C14* locus largely reduces egg production but has little effect on galling [9]. Further, while the main effects of each QTL appeared to serve as the major genetic basis in conferring resistance for both galling and egg production phenotypes, additive x additive epistatic interaction was important in suppressing nematode egg production, resulting in a near-immunity to infection when both QTLs are present [9]. These studies support earlier histo-pathological observations indicating that a two-stage defense mechanism was responsible for resistance in Auburn 623 RNR. The first stage involved suppressing the development of giant cells at 6 days post penetration and the second stage involved reducing the egg production of *M. incognita* females at 24 days post penetration [10, 11].

The presence of two or more unlinked resistance loci with different mechanisms in the Auburn 623 RNR source has complicated elucidating the mode of inheritance and determining the genetic effects of each resistance gene. For example, Zhou et al. [12] reported that RKN resistance in the Auburn 623 RNR source involved a partially dominant and a recessive gene. However, McPherson et al. [13] suggested that resistance in the Auburn 623 RNR source was due to a dominant and an additive gene, and Zhang et al. [14] concluded that resistance was controlled by at least two additive genes. To better determine the genetic effects of each resistance locus and to investigate the hypothesis of independent resistance genes controlling plant responses to suppression of root galling and nematode reproduction in this resistance source would require testing a genetic population segregating for only a single resistance locus.

Host-plant resistance to root-knot nematodes was first identified in *Lycopersicum peruvianum* Mill., a wild relative of cultivated tomato [15] where a single dominant *Mi* gene conferred resistance to three major root-knot nematodes *M. arenaria, M. incognita* and *M. hapla*. Further molecular characterization of the *Mi* gene revealed that it encodes a protein with nucleotide-binding site leucine-rich repeat (NBS-LRR) motifs [16] and triggers a hypersensitive response in the host plant. Based on the amino acid motifs and their membrane spanning domains [17], disease resistance genes in plants are grouped into eight classes and a majority of these genes are characterized by the presence of Leucine Rich Repeat (LRRs) motifs. The several classes of LRR-containing proteins that exist in plants have diverse overall structures and functions. They provide an early warning system for the presence of potential pathogens and activate protective immune signaling in plants [18, 19]. In addition, they act as a signal amplifier in the case of tissue damage, establishing symbiotic relationships and affecting developmental processes [20].

In this study, we developed a genetic population segregating only for the *qMi-C14* resistance locus. Our objectives were to: (1) develop and map new SSR markers to construct a dense linkage map near the *qMi-C14* locus, (2) estimate the genetic effect of *qMi-C14* on RKN resistance and (3) predict putative candidate genes responsible for the *qMi-C14* resistance by identifying genes that encode for LRR-containing proteins within the QTL interval.

## Methods

### Population development

The mapping population was derived from an interspecific cross between the highly resistant Upland cotton line M-120 RNR, and an RKN susceptible *G. barbadense* cultivar Pima S-6 as described by Shen et al. [7]. A total of 1252 F_2_ individuals were planted in a greenhouse and screened with previously published SSRs mapped to chromosomes 11 and 14 [6, 7, 9]. Seven F_2_ individuals that were completely heterozygous at the *qMi-C14* locus on Chromosome 14 as determined by five previously mapped co-dominant SSR markers (CIR246, JESPR156, UGT045, CGR5668, and STV30) [9] but were homozygous for the Pima S-6 allele at the *qMi-C11* locus as determined by CIR069 and CIR316 on chromosome 11 (for absence of resistance allele) were identified. All seven plants were self-pollinated to yield a genetic population consisting of 513 plants segregating only for the *qMi-C14* locus.

### Phenotypic screening and data analysis

The mapping population was planted in a greenhouse along with ten plants each of the parental lines, M-120 RNR and Pima S-6, as resistant and susceptible checks, respectively. The screening method to determine the level of resistance to RKN has been described previously by Shen et al [7]. One hour prior to inoculation, nematode eggs were collected by agitating infected eggplant roots (*Solanum melongena* L.) in 0.5 % sodium hypochlorite solution for two minutes [21]. Three week old plants were inoculated with approximately 8000 eggs of *M. incognita* race 3 (approximately 450 eggs 150 cm^-3^ soil). Inoculum was distributed into two holes about 2.5 cm deep in soil on opposite sides of the seedling and the holes were covered with soil after inoculation. The soil temperatures varied between 17 °C and 28 °C. Plants were evaluated for the amount of *M. incognita* reproduction at 56 to 58 days after inoculation. Phenotypic data collected included root galling index (RGI), total number of eggs extracted, and the number of eggs per gram of fresh root. Roots were washed free of soil, evaluated for galling, weighed, cut into 5-cm pieces, and agitated in a 1 % v/v sodium hypochlorite solution in a 1-liter flask for four minutes [21]. Eggs were collected and rinsed with tap water on nested 150- over 25-μm-pore sieves. Root galling index (RGI) was evaluated using the 0 to 10 scale where 0 = no galling, 1 = 1–10 % of the root system galled, 2 = 11–20 % of the root system galled, up to 10 = 91–100 % of the root system galled [22]. Roots of each plant were harvested and weighed immediately prior to extraction of eggs. Eggs were extracted from the whole root system and counted. The mean number of eggs per gram of fresh root (eggs/g of root) was evaluated in order to standardize egg counts.

Statistical parameters describing the population for each phenotypic trait were calculated using SAS (SAS, ver. 9.3). Fisher’s Least Significant Difference (LSD) test was used to compare phenotypic means for all traits between mapping parents. Data for total egg count and eggs per gram of root weight were Log_10_ (x + 1) transformed to achieve a normalized distribution for QTL analysis.

### SSR mining and primer design

He et al. [9] and Gutierrez et al. [8] indicated that the RKN resistance locus on chromosome 14 is flanked by BNL3545 and CGR5668; therefore, we used the sequences from which these two SSRs (available at www.cottongen.org) to BLAST search the genome sequence of *G. raimondii* at www.phytozome.net (version 9.1). The physical location of the *qMi-C14* locus was found to be on the distal end of *G. raimondii* chromosome 05. More than 4 Mbp of sequence from the distal end of this chromosome was downloaded in fasta file format, divided into two equal segments of 2Mbp each, and scanned for short repeating units using a web based microsatellite repeat finder (insilico.ehu.es/mini_tools/microsatellites). Search parameters were: minimum number of repeats = 5, minimum repeat unit length = 2, maximum repeat unit length = 10. Fifty microsatellites that were between 10–25 repeats long were selected; these microsatellites are fairly uniformly distributed along the 4 Mbp region. Three hundred base pairs on each side of each microsatellite were selected for primer development using the web based BatchPrimer3 software [23]. Primers were 20–22 nt long and designed to amplify 150–200 bp PCR products at melting temperatures between 50–55 °C. Primers were commercially synthesized by Eurofins MWG Operon (Huntsville, AL).

PCR reaction conditions were slightly modified from a previously described protocol [24]. PCR amplification was performed in a PTC-100 or PTC-200 thermocycler (MJ Research Inc.). A 10 μl reaction contained 10 ng of template DNA, 0.5 μM primer mix, 100 μM dNTPs, 1.5 mM MgCl_2_, 3U of DNA polymerase, and 1X reaction buffer (100 mM Tris-HCl, pH 8.3, 500 mM KCl). The cycling conditions for PCR were 94 °C for 3 min, followed by 35 cycles of 94 °C for 1 min, 55 °C for 1 min, and 72 °C for 1.2 min. After the last cycle, reactions were incubated at 72 °C for 6 min before cooling to 4 °C. The amplified PCR products were separated by electrophoresis on a 10 % w/v non-denaturing polyacrylamide gel (PAGE) and visualized by staining with silver nitrate following published procedures [25].

### Linkage map construction and QTL mapping

Procedures for linkage map development and QTL analysis were as described by He et al. [9]. Briefly, all markers were first tested for their ability to detect polymorphism between the M-120 RNR and Pima S-6 parents. Primers that detected clear polymorphism between parents were utilized to genotype the entire mapping population. Linkage groups were constructed using the Mapmaker/EXP 3.0 [26] software. Linkage group assembly was done using the ‘group’ command with a LOD score of 3.0 and a maximum recombination fraction of 30 cM. Recombination units were converted into genetic distances by using the Kosambi mapping function [27] with the “error detection” command on. New markers were added to the framework using the ‘try’ and ‘compare’ commands. The final order of markers on a linkage group was confirmed using the ‘ripple’ command.

Detection of QTLs and estimation of various genetic parameters were performed by Composite Interval Mapping (CIM) function implemented in the software WinQTL Cartographer version 2.5 [28]. Likelihood ratio (LR) threshold values (α = 0.05) for declaring the presence of QTLs were estimated after 1000 permutations [29]. Mapping was performed at 1 cM walk speed in a 10 cM window using five background cofactors selected via forward-backward stepwise regression. QTLs were defined by one-LOD likelihood intervals on either side of the peak position.

### Identification of potential candidate genes

The reference genome sequence of the D genome diploid *G. raimondii* and the assembled D_t_ subgenome of the tetraploid *G. hirsutum* were downloaded from CottonGen (http://www.cottongen.org) along with their GFF3 gene annotation files. The physical region between markers BNL3545 and STV030 was identified in both genome sequences and scanned for annotated genes using the Multiple Sequence Comparison by Log-Expectation software [30]. Genes with Leucine Rich Repeat (LRR) N-terminal domains were shortlisted as potential candidate genes that may be involved in a nematode resistance. Protein sequences encoded by these genes were further used for blastp (protein-protein blast) search to identify protein homologs in the protein database of GenBank. A neighbor joining method was utilized to construct the phylogenetic tree using the MEGA 6.0 software [31].

### Identification of single nucleotide variations

Genomes of an RKN susceptible cotton line, Acala ‘Maxxa’ (SRA061309), and an RKN resistant line, GA120R1B3 (SRA068148), were sequenced at 82x and 30x read depth respectively by Illumina sequencing of paired-end libraries [32]. Using the cotton D-genome sequence as reference [33], short reads were aligned using the Burrows-Wheeler Aligner (BWA) [34]. Single nucleotide polymorphism (SNPs) between candidate gene alleles in RKN resistant and susceptible lines were called with Samtools/bcftools [35, 36] using reads with mapping quality >30 and base quality >30 [32].

## Results

### Population biometrical parameters

**Fig. 1:**
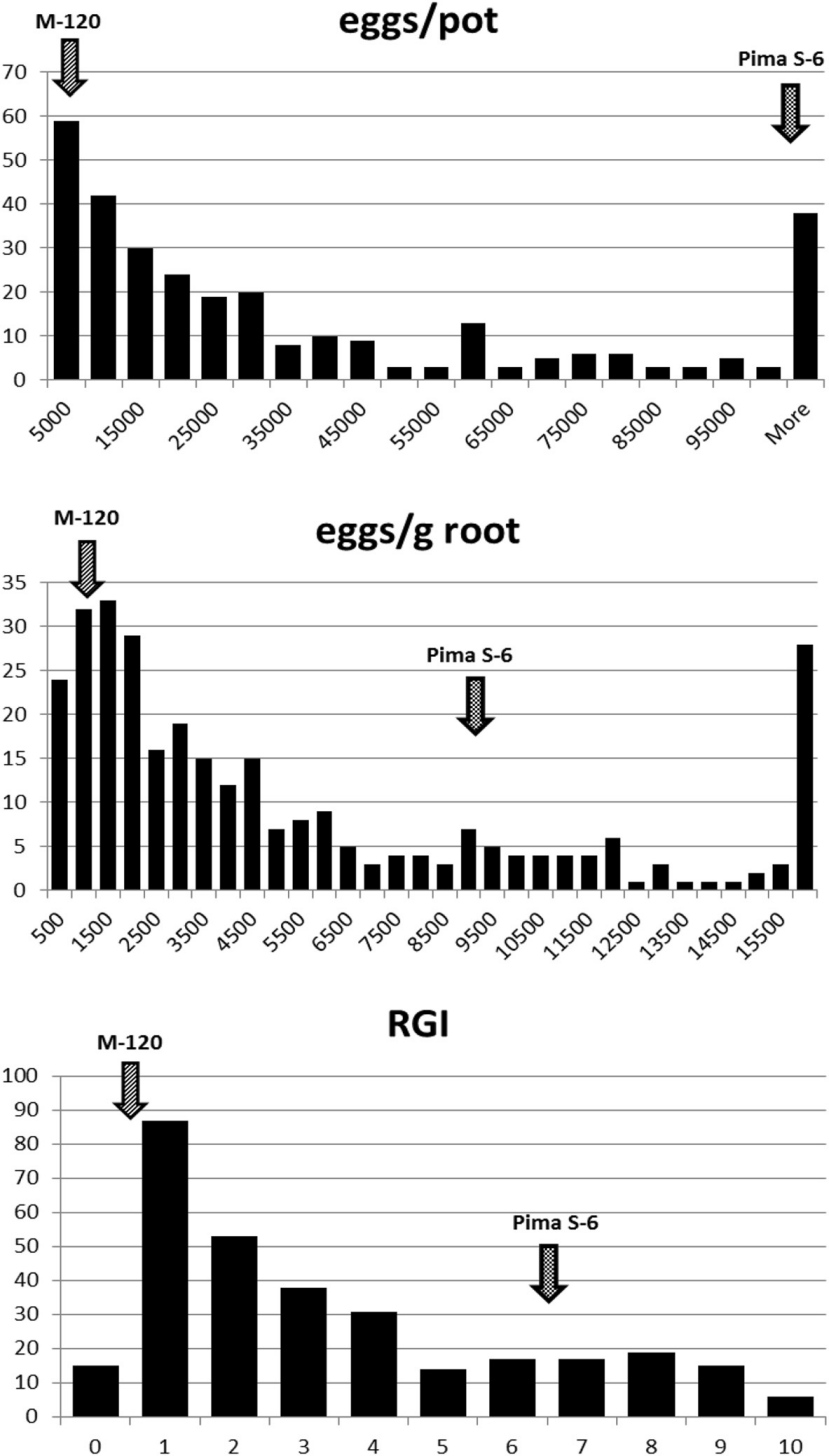
Phenotypic distribution of Root Galling Index (RGI) and egg production in an F_2_ population segregating for the *M. incognita* resistance QTL *qMi-C14*

The distribution of resistance reaction phenotypes for the parents and the mapping population is presented in Fig. 1. The resistant parent, M-120 RNR had significantly (*P* < 0.001) lower galling, total eggs, and eggs g^-1^ root than the susceptible parent, Pima S-6. A non-normal distribution was observed for total eggs and eggs g^-1^ root (skewness > 1.0) with greater number of individuals skewed towards the M-120 RNR parent, suggesting a dominant nature of the gene(s) underlying RKN resistance on chromosome 14. A strong correlation was observed between eggs pot^-1^ and eggs g^-1^ root (*r* = 0.83, *p < 0.0001*). However, RGI had a weak correlation with eggs pot^-1^ (*r* = 0.19, *p < 0.01*) and eggs g^-1^ root (*r* = 0.18, *p < 0.05*), suggesting that RGI and egg production were genetically independent but together contribute toward RKN resistance in the mapping population. Root weight was not correlated with RGI or eggs g^-1^ root but was moderately correlated with eggs pot^-1^ (*r* = 0.36, *p < 0.0001*).

### New SSR marker development and mapping

A total of 801 microsatellites were found on the 4 Mb region of *G raimondii* chromosome 05 between the two SSR markers BNL3545 and CGR5668, thought to contain *qMi-C14*, 405 microsatellites were identified in the first 2 Mb and 396 in the next 2 Mb region. The distribution of di, tri, tetra, penta and hexa repeats on the two regions is presented in Additional file 1: Figure S1. Of the 801 microsatellites identified, 624 (77.9 %) di-nucleotide repeats and 135 were tri-nucleotide repeats with unit copy numbers from 5–28. Forty-five SSRs occurring at approximately uniform intervals were selected to fine-map the region between BNL3545 and CGR5668. A polymorphism survey found 27 (60 %) SSRs to be polymorphic between M-120 RNR and Pima S-6, with 12 (27 %) being monomorphic and 6 (13 %) failing to amplify.

**Fig. 2:**
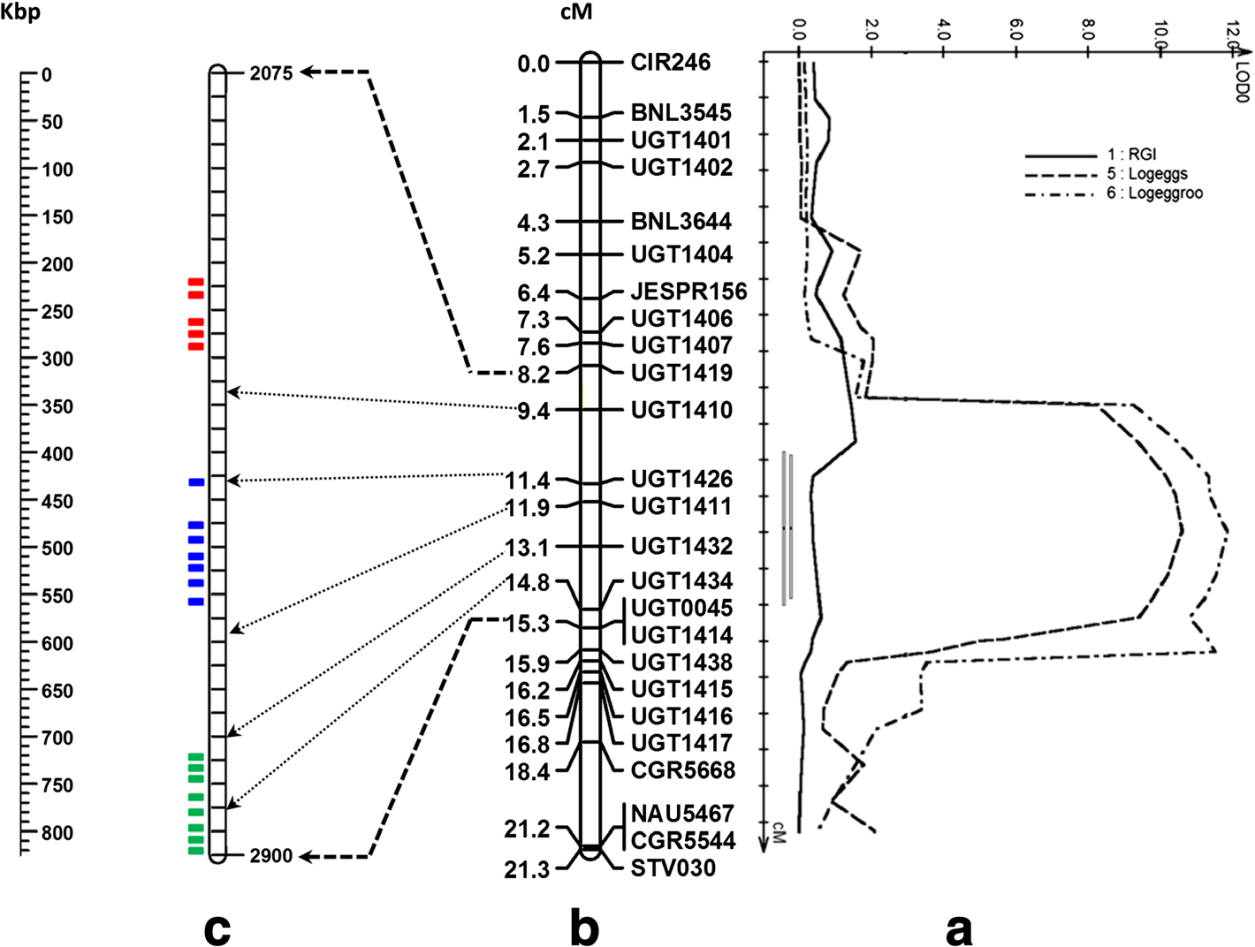
Genetic and physical position of *qMi-C14* on chromosome 14. **a** LOD profile of the QTL region affecting RKN egg production; **b** Linkage map of the telomeric region; and **c** Physical location of the three clusters of candidate genes, identified with different colors

Figure 2 shows the localized linkage map of the *qMi-C14* region on chromosome 14. A total of 25 loci were mapped to this region spanning 21.3 cM (average distance of 0.85 cM between markers), 16 of which were newly developed SSR markers and 9 were previously reported by He et al. [9]. Eleven of the 27 polymorphic markers did not produce scorable fragments or did not map on chromosome 14, and hence were not pursued further in this study. Forward and reverse primer sequences of the 16 new SSR markers are presented in Additional file 2: Table S1. No significant segregation distortion was observed for any of these markers.

### Association analyses

Because these phenotypes displayed a non-normal distribution, log transformed data for total eggs and eggs g^-1^ of root phenotypes were used for further analysis. Composite interval mapping detected a major QTL for total eggs and eggs g^-1^ root near the marker UGT1432, with the allele from the resistant parent M-120 RNR increasing nematode resistance for all phenotypes. The QTL peak for both phenotypes was located at the 12.9 cM position with a LOD value of 10.7 and 11.8, respectively. These QTLs explained up to 21.5 % and 24.6 % of phenotypic variation for total eggs and eggs g^-1^ root, respectively. The likelihood interval, circumscribed by a one LOD score drop on either side of the peak, covered 3.9 cM and was flanked by markers UGT1426 and UGT1414 (Fig. 2). The d/a ratio was 0.1 for both phenotypes, indicating that the gene action for this QTL is additive. Interestingly, there was no significant association with RGI suggesting that this region has no effect on suppressing root galling.

**Fig. 3:**
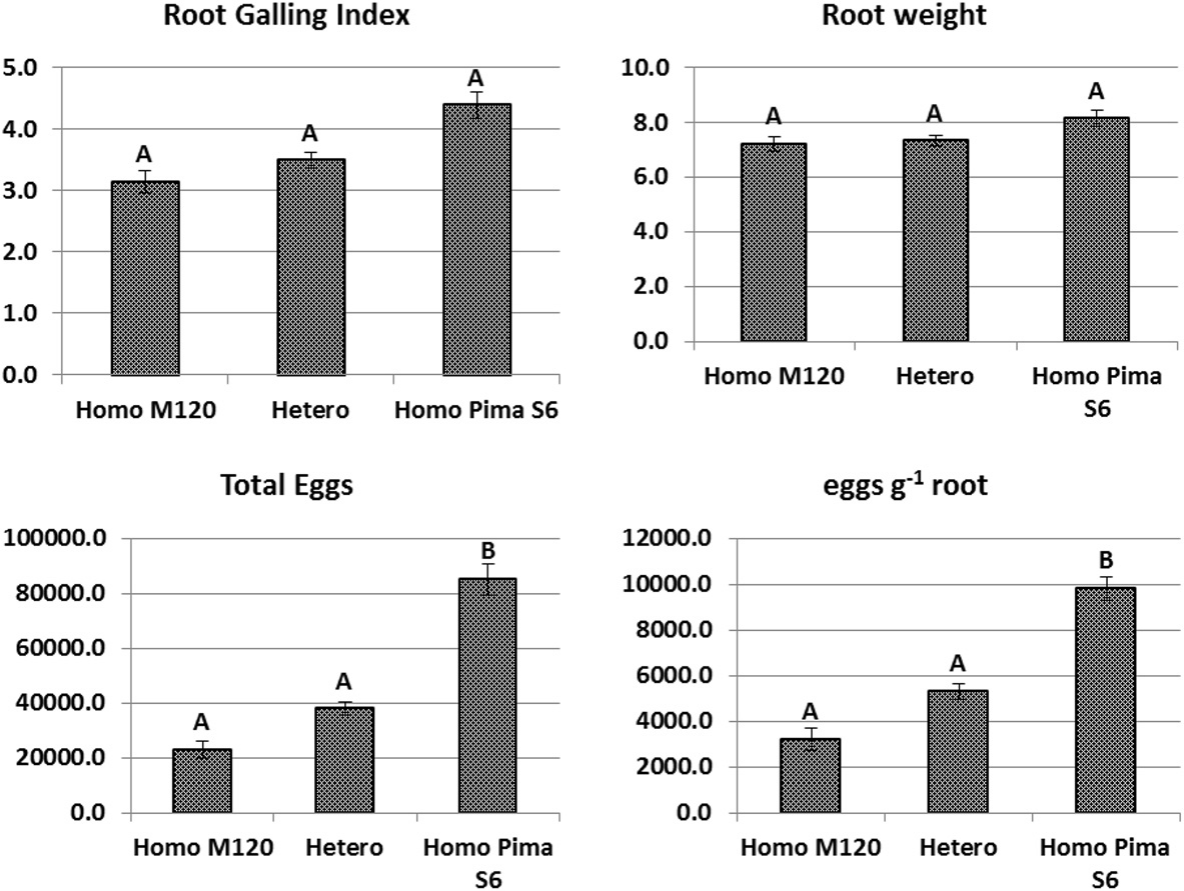
Mean difference between the three genotypic classes in the BNL3545 -- STV030 interval. Means were compared (*P < 0.001*) between individuals completely homozygous for the M-120 allele (Homo M120), completely homozygous for Pima S-6 alleles (Homo Pima S6) and completely heterozygous (Hetero)

To more precisely evaluate the genetic effect of *qMi-C14*, all individuals homozygous for the M-120 RNR alleles, homozygous for the Pima S-6 alleles and heterozygous for the entire region between BNL3545 and STV030 that flanks the *qMi-C14* locus, were selected for further analysis (Fig. 3). In total, 50 individuals were completely homozygous for the M-120 RNR allele, 55 were completely homozygous for Pima S-6 alleles and 119 were completely heterozygous for the region between BNL3545 and STV030. Differences in phenotypic means for total eggs and eggs g^-1^ root between the two homozygous classes were highly significant (*P < 0.001*). The difference in phenotypic means for total eggs and eggs g^-1^ root between the heterozygous class and the Pima homozygous S-6 class was significant, but no significant difference was observed between the heterozygous and the M120 RNR homozygous class (Fig. 3). These results suggest that although the d/a ratio indicates that the gene action was additive, this locus may have dominance effects. No significant difference was observed between the means of the three genotypic classes for RGI or root weight.

### Potential candidate genes

**Fig. 4:**
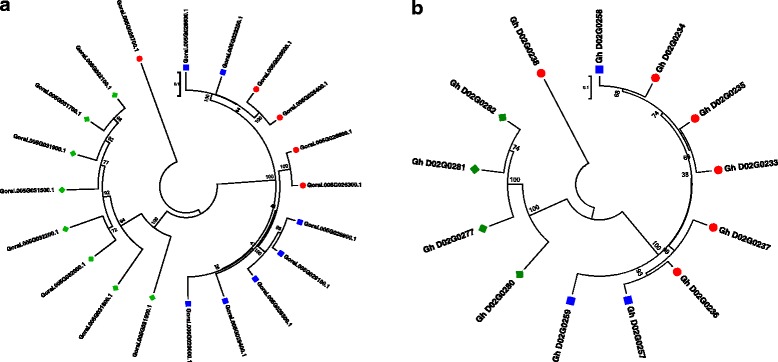
Phylogenetic relationships among the candidate genes with LRR motifs near the *qMi-C11* locus, from *G. raimondii* (panel **a**) and *G. hirsutum* (panel **b**). Red dots, blue squares and green diamond represent genes from Group I, II, and III, respectively

More than 133 annotated genes were identified in the physical region between the markers BNL3545 and STV030 (www.phytozome.net), including 20 in the *G. raimondii* genome and 13 in the orthologous region from the D_t_ subgenome of *G. hirsutum* (Table 1) that contain leucine rich repeat (LRR) N-terminal domains. Based on the *G. raimondii* physical map, the LRR candidate genes were distributed into three groups (Fig. 2). Most of the LRR genes within each group shared a high level of homology as indicated by their clustering in the phylogenetic tree drawn from multiple alignments of the protein coding regions of these genes (Fig. 4). For example, all eight genes in Group III fall into a single clade in the phylogenetic tree. The genes in Group I and II were more similar to each other with two genes in Group I clustering with four genes in Group II, and two genes each in Group I and II forming the third clade in the phylogenetic tree. Finally, the gene Gorai.005G026700 from Group I appears to be the most diverse as it does not cluster with the other groups. The 13 LRR genes identified in the D02 chromosome of *G. hirsutum* also showed a similar distribution on the physical map and clustering pattern in the phylogenetic tree (Fig. 4). A blast search indicated that all genes except Gorai.005G031600 in *G. raimondii* showed a high degree of similarity (*E-value* = 0) with at least one of the genes found in *G. hirsutum*. Interestingly, Gorai.005G031600 did not yield any hit on the D02 chromosome of the *G. hirsutum* D_t_ sub-genome, suggesting that it may have been lost from the tetraploid genome.

**Table 1:** Physical locations of genes with Leucine Rich Repeat (LRR) domain between SSR markers BNL3545 and STV030 on chromosome 5 of *G. raimondii* and chromosome D02 of *G. hirsutum*

Gene name	Physical location	Group
Gorai.005G026300	2297846 - 2301061	I
Gorai.005G026400	2313103 - 2316975	I
Gorai.005G026500	2345315 - 2348063	I
Gorai.005G026600	2349170 - 2352262	I
Gorai.005G026700	2361518 - 2365096	I
Gorai.005G028600	2501120 - 2504324	II
Gorai.005G028900	2553716 - 2557308	II
Gorai.005G029000	2565325 - 2569455	II
Gorai.005G029100	2585224 - 2589560	II
Gorai.005G029200	2596425 - 2600231	II
Gorai.005G029300	2613108 - 2617044	II
Gorai.005G029400	2632315 - 2635858	II
Gorai.005G031500	2804242 - 2806833	III
Gorai.005G031600	2810110 - 2813150	III
Gorai.005G031700	2819656 - 2825365	III
Gorai.005G031800	2833071 - 2838460	III
Gorai.005G031900	2852829 - 2859789	III
Gorai.005G032000	2870255 - 2876978	III
Gorai.005G032100	2880034 - 2881241	III
Gorai.005G032200	2898234 - 2905491	III
Gh D02G0233	2749065 - 2751671	I
Gh D02G0234	2757983 - 2759275	I
Gh D02G0235	2766873 - 2769728	I
Gh D02G0236	2780046 - 2781380	I
Gh D02G0237	2787974 - 2791318	I
Gh D02G0238	2800055 - 2803296	I
Gh D02G0257	2942631 - 2946086	II
Gh D02G0258	2962327 - 2965794	II
Gh D02G0259	2979373 - 2983290	II
Gh D02G0277	3219359 - 3311044	III
Gh D02G0280	3391502 - 3393436	III
Gh D02G0281	3410121 - 3412647	III
Gh D02G0282	3466152 - 3467675	III

Gene name with prefix ‘Gorai’ are from *G. raimondii* and those with prefix ‘Gh’ are from *G. hirsutum*

### SNP between RKN resistant and susceptible alleles

Sequences of protein coding regions (CDS) and untranslated regions (5′ and 3′UTRs) of 59 genes between Gorai.005G026300 and Gorai.005G032200 (about 600 Kbp), including 20 that contain LRR motifs, were compared between Acala ‘Maxxa’ (RKN susceptible) and GA120R1B3 (RKN resistant) lines. Twenty-five SNPs were identified in 11 genes that differentiate the RKN resistant line from the susceptible Acala Maxxa (Table 2). SNP alleles in coding regions of four LRR genes in the RKN resistant line GA120R1B3 – Gorai.005G026500, Gorai.005G028600, Gorai.005G029200 and Gorai.005G029400 – resulted in changes in amino acids relative to Acala Maxxa (Table 2).

**Table 2:** Details of non-synonymous SNPs that differentiate genes from GA120R1B3 (RKN resistant) and Acala Maxxa (susceptible) lines in the QTL region flank by Gorai.005G026300 and Gorai.005G032200

Gene ID	Position	Strand	Ref allele	Susceptible allele	Resistant allele	Ref aa	Susceptible aa	Resistant aa	SNP location	Pfam	Annotated function
Gorai.005G026500	2345728	+	T	T	G	F	F	L	CDS	PF08263,PF00560,PF13855	receptor like protein 7
Gorai.005G027700	2415658	-	T	T	G	.	.	.	3-UTR	PF02826,PF05221	S-adenosyl-L-homocysteine hydrolase
Gorai.005G028100	2458792	-	T	T	C	.	.	.	3-UTR	PF00076	glycine-rich RNA-binding protein 2
Gorai.005G028100	2459025	-	A	A	G	.	.	.	3-UTR	PF00076	glycine-rich RNA-binding protein 2
Gorai.005G028100	2460937	-	A	A	T	.	.	.	5-UTR	PF00076	glycine-rich RNA-binding protein 2
Gorai.005G028500	2499549	+	C	C	T	.	.	.	3-UTR	PF05653	Protein of unknown function (DUF803)
Gorai.005G028600	2502813	+	T	T	A	S	S	R	CDS	PF08263,PF00560,PF13855	receptor like protein 7
Gorai.005G028600	2502817	+	G	G	A	D	D	N	CDS	PF08263,PF00560,PF13855	receptor like protein 7
Gorai.005G029200	2599856	+	G	G	T	K	K	N	CDS	PF08263,PF13855	receptor like protein 6
Gorai.005G029200	2599857	+	A	A	T	I	I	F	CDS	PF08263,PF13855	receptor like protein 6
Gorai.005G029400	2632994	+	T	T	C	V	V	A	CDS	PF08263,PF13855	receptor like protein 6
Gorai.005G029400	2634053	+	G	G	C	S	S	T	CDS	PF08263,PF13855	receptor like protein 6
Gorai.005G029600	2657576	+	G	G	A	A	A	A	CDS	PF00119	ATPase, F0 complex, subunit A protein
Gorai.005G029600	2657842	+	G	G	A	R	R	Q	CDS	PF00119	ATPase, F0 complex, subunit A protein
Gorai.005G030200	2695818	-	T	T	A	.	.	.	3-UTR	PF01564,PF08241	S-adenosyl-L-methionine-dependent methyltransferases superfamily protein
Gorai.005G030800	2750823	-	G	G	A	.	.	.	5-UTR	PF05856	protein binding
Gorai.005G031000	2760819	+	C	C	T	.	.	.	5-UTR	PF02892,PF02902,PF05699	BED zinc finger ;hAT family dimerisation domain
Gorai.005G031000	2761040	+	A	A	C	.	.	.	5-UTR	PF02892,PF02902,PF05699	BED zinc finger ;hAT family dimerisation domain
Gorai.005G031000	2761073	+	A	A	G	.	.	.	5-UTR	PF02892,PF02902,PF05699	BED zinc finger ;hAT family dimerisation domain
Gorai.005G031000	2761385	+	C	C	A	R	R	S	CDS	PF02892,PF02902,PF05699	BED zinc finger ;hAT family dimerisation domain
Gorai.005G031000	2761993	+	G	G	C	K	K	N	CDS	PF02892,PF02902,PF05699	BED zinc finger ;hAT family dimerisation domain
Gorai.005G031000	2761995	+	C	C	G	T	T	S	CDS	PF02892,PF02902,PF05699	BED zinc finger ;hAT family dimerisation domain
Gorai.005G031000	2762049	+	A	A	G	E	E	G	CDS	PF02892,PF02902,PF05699	BED zinc finger ;hAT family dimerisation domain
Gorai.005G031000	2768718	+	A	A	G	N	N	D	CDS	PF02892,PF02902,PF05699	BED zinc finger ;hAT family dimerisation domain
Gorai.005G031000	2768729	+	A	A	T	P	P	P	CDS	PF02892,PF02902,PF05699	BED zinc finger ;hAT family dimerisation domain

## Discussion

Transgressive segregation for RKN resistance from the Auburn 623RNR cotton germplasm is due to the effects of pyramiding of at least two QTLs inherited from different parents; each individually confers moderate resistance, although the two QTLs interact epistatically to confer near-immunity. The *qMi-C11* and *qMi-C14* loci map to chromosome 11 and 14, respectively [8, 9]. Study of these loci shows that two phenotypes commonly used to measure resistance reaction to RKN, specifically galling index and egg production, could be partly genetically independent, and the *qMi-C11* and *qMi-C14* loci may provide resistance via different mechanisms [8, 9]. Full characterization of q*Mi-C11* and q*Mi-C14* will advance our understanding of defense mechanisms against parasitism from RKN, elucidate host-pathogen interactions at the molecular level, and facilitate their use in cotton improvement.

The genetic population used in the current study, consisting of 513 F_2:3_ plants, was developed using F_2_ plants that were heterozygous (and therefore segregating) at the *qMi-C14* locus but homozygous for the susceptible parent allele at the *qMi-C11* locus. Without the confounding effects from the *qMi-C11* QTL, our data confirmed prior QTL analyses suggesting that the *qMi-C14* locus had a highly significant effect on egg production but little or no effect on root galling (Fig. 3). The estimated main genetic effects for this locus was 21.5 to 24.5 % PV explained, lower than the 45 to 47 % PV explained in our earlier study when both QTLs were segregating in the population [9]. The suppression of egg production with *qMi-C14* alone appears to be lower in the absence of the *qMi-C11* locus, further supporting the hypothesis that the combination of both QTLs with different modes of action provides the genetic basis for transgressive segregation in the Auburn 623RNR germplasm [8, 9]. Similar transgressive resistance to root-knot nematode resulting from epistatic interaction have been reported in cotton. For example, Wang et al. [37] reported that the gene *RKN2* from the susceptible *G. barbadense* cultivar Pima S-7 can significantly enhance the level of resistance to RKN in the Acala NemX resistance source. In addition to cotton, interlocus interactions among main effect resistance QTLs have been observed in pepper [38] and soybean [39], suggesting that epistasis is common and constitutes a major component of host-plant resistance to pest and disease pathogens.

The reference genome sequence of the diploid *G. raimondii* [33] and draft sequence of the tetraploid Upland cotton *G. hirsutum* [40, 41] have provided the cotton community an unprecedented resource for genome analysis. In this study, we were able to narrow the confidence interval for *qMi-C14* to a 3.9 cM region and identify 20 candidate resistance genes with conserved LRR domains clustering in three groups (Fig. 4). This is not surprising as NBS-LRR genes are frequently clustered in plant genomes, and can contain genes conferring resistance to diverse pathogens, thereby acting as reservoirs of genetic variation for resistance specificities [17]. More importantly, of the six RKN resistance genes that have now been cloned in plants, all fall into a family of genes with the classic LRR motif typical of disease resistance genes (R-genes) [42]. Not all genes may be expressed at a given time but specific R genes may be triggered by the avirulence (*Avr*) factor of the invading pathogen, and result in activation of a gene-for-gene resistance mechanism. Since a majority of disease resistance genes are comprised of arrays of hypervariable potential ligand-binding sites, inter-allelic recombination within these arrays along with unequal recombination may be the primary mechanism behind rapid evolution of *R* genes and their clustering in the genome [43].

To further investigate the role of these LRR genes in disease resistance, we performed a Blast search of GenBank to identify annotated genes from species for which the genome sequence is available. Except for the gene Gorai.005G026700 and its tetraploid genome counterpart Gh D02G0238 that showed high similarity with a *Theobroma cacao* leucine-rich repeat protein kinase family protein isoform (accession No. XP_007034190.1), all other candidate genes from cluster I and II (Group I and II on the cladogram) showed high similarity with a *Theobroma cacao* Verticillium wilt disease resistance-like protein (up to 82 % sequence similarity to accession XM_007034192.1). Genes in cluster III showed high homology with a *Theobroma cacao* receptor like protein (accession XM_007010859.1). This observation is not uncommon - as noted in earlier studies, *R* genes in a disease resistance cluster encode protein motifs that are components of signal transduction systems including genes coding for protein receptors and protein kinases. For example, the *Xa21 R* gene cluster in rice is composed of more than eight LRR and protein kinase genes while the *M* locus in flax and *RPP5* locus in Arabidopsis are comprised of genes with NBS-LRR and Toll-interleukin like protein receptor domains [44]. Molecular studies have shown that some *R* genes in a disease resistance cluster share some degree of sequence similarity with other genes but are functionally divergent. For example, *Prf*, an NBS-LRR *R* gene, is located within a cluster of five protein kinase homologs (*Pto*). Both *Prf* and *Pto* genes are required for resistance to *Pseudomonas syringae* pv. *tomato* [44], suggesting that resistance is conferred by a haplotype rather than an individual gene.

Among 20 positional candidate LRR genes, sequencing of the QTL interval of the resistant germplasm GA120R1B3 (derived from Auburn 623RNR) revealed only four to have non-synonymous mutations in the coding region (Table 2), suggesting these to be more probable candidates than the others. The observation that the SNP containing LRR gene Gorai.005G026500 belongs to cluster-I and Gorai.005G028600, Gorai.005G029200, and Gorai.005G029400 belong to cluster-II implicates one of these two clusters in RKN resistance. Such non-synonymous mutations in the coding region are likely to have caused changes in the structure and function of the encoded protein, but the effects of these mutations are currently unknown.

Although no nematode resistance genes have been cloned via map-based cloning in cotton, a polypeptide called *Meloidogyne* Induced Cotton or *MIC* protein has been observed to be differentially expressed in root galls of resistant and susceptible cotton plants [45]. Further analyses on the *MIC* protein led to the identification of the *MIC-3* gene family with at least 15 MIC-like cDNAs that encode cotton-specific pathogenesis-related proteins [46, 47]. Overexpression of *MIC*-3 in a RKN susceptible cotton line showed 60–70 % reduction in RKN egg production when compared to non-transgenic controls [40]. Further, overexpression of *MIC*-3 does not affect RKN-induced root galling. Therefore, the *MIC* gene family produced a similar phenotypic effect as *qMi-C14*, reducing nematode egg production but not root galling. However, our BLAST search against the *G. hirsutum* genome database using the MIC sequence (Accession No. AY072783) showed no *MIC*-like genes in or near the *qMi-C14* region, precluding *MIC* family members as candidate genes for this QTL. Nonetheless, given that *MIC* expression and effects are strongly correlated with RKN resistance, it is possible that this gene family represents a type of pathogenesis-related gene in which the effect is at the ‘end-point” in the signaling cascade that is regulated by gene(s) annotated in the *qMi-C14* locus.

From a practical breeding perspective, until the causal gene for *qMi-C14* is identified, it might be prudent to simultaneously target all three clusters of R genes from the resistant parent to ensure horizontal and possibly stable RKN immunity of new cultivars. Tightly linked markers are therefore important for detecting recombination events that may result in segregation of these R gene clusters, as well as to identify the zygosity of selected plants during marker-assisted selection. Our results further point to the dominant nature of this QTL as indicated by non-significant differences between the heterozygous and homozygous classes of resistance alleles. This scenario can potentially render selection based solely on phenotypic observation less effective in cotton breeding populations, and may have contributed to difficulties in fixing resistance alleles during the development of the highly resistant germplasm line GA 120R1B3 [48].

## Conclusion

In this study, we validated an earlier observation that the qMi-C14 resistance locus mostly suppress egg production but has little effect on galling, further supporting the hypothesis that the effectiveness of RKN resistance from Auburn 623RBR is due to synergistic effects of two different mechanisms of RKN resistance. In addition, we have delimited qMi-C14 to a physical contig of about 2.3 Mb and identified 20 positional candidate LLR genes, including four of which containing non-synonomous mutation in the coding region. The candidate genes identified warrant functional studies that will help in identifying and characterizing the actual qMi-C14 defense gene(s) against root-knot nematodes.

## Abbreviations

LRR, leucine rich repeat, LOD, likelihood of odds ratio, NBS, nucleotide binding site, QTL, quantitative trait locus, RKN, root-knot nematodes, RNR, root-knot nematode resistant, SSR, simple sequence repeats

## Additional files

Additional file 1: Figure S1. Distribution of types of SSRs in the 4 Mb telomeric region of chromosome 5 of *G. raimondii. (DOCX 30 kb)*
Additional file 2: Table S1. Primer sequences of newly mapped SSRs developed from *G. raimondii* sequences. (DOCX 14 kb)
