# Supplementary material for: Fine mapping and identification of candidate genes for a QTL affecting Meloidogyne incognita reproduction in Upland cotton
Source: BMC Genomics. 2016 Aug 8;17:567. doi: 10.1186/s12864-016-2954-1 (PMC4977665; doi:10.1186/s12864-016-2954-1)
Supplement: Additional file 1: Figure S1. — Distribution of types of SSRs in the 4 Mb telomeric region of chromosome 5 of G. raimondii. (DOCX 30 kb) [file 12864_2016_2954_MOESM1_ESM.docx]

Fig S1. Distribution of types of SSRs in the 4Mb telomeric region of chromosome 5 of *G. raimondii.*
