# Supplementary material for: Fine mapping and identification of candidate genes for a QTL affecting Meloidogyne incognita reproduction in Upland cotton
Source: BMC Genomics. 2016 Aug 8;17:567. doi: 10.1186/s12864-016-2954-1 (PMC4977665; doi:10.1186/s12864-016-2954-1)
Supplement: Additional file 2: Table S1. — Primer sequences of newly mapped SSRs developed from G. raimondii sequences. (DOCX 14 kb) [file 12864_2016_2954_MOESM2_ESM.docx]

Table S1. Primer sequences of newly mapped SSRs developed from *G. raimondii* sequences.

| SSR Name | FORWARD | REVERSE |
| --- | --- | --- |
| UGT1401 | ACTTTTCCACGTCATTTACAA | AGCTTCAAAATTAGACCGATT |
| UGT1402 | GTTTGATTTCCGAGAAAATGT | GGCCCTAATGCTGTACTTTAC |
| UGT1404 | TCGAGTGAACCTATTGAAAGA | GTTGATTAACCCATGAAAGGT |
| UGT1406 | CGATTTAAGTCCATGAATGAG | ACTCCGAATCTCGAAAAATTA |
| UGT1407 | CCTATTCGAAACTACATATACAA | TCTTGGTTTTAATCAAAGGTATG |
| UGT1410 | TCCTGATTCAAACGATGTATT | TCATTTGGATGAATTGTAACG |
| UGT1411 | GCACCGTGGGTTAAAATTAG | TGACTCACCTAATTGGATGTC |
| UGT1414 | ACGCTCGTATTTTGAAACTTA | AACTGAAACAAACGCTACAAC |
| UGT1415 | CCTCTCTTCACGCCTTACTAT | TATCGTTGGATTGAGAAGTTG |
| UGT1416 | GATATAACTTATCACGGCATCC | AAAAAGACAGTCATTCCACAA |
| UGT1417 | CAGACACAGACACAGACACAG | AAGTCTCAACCAGGCATAAAT |
| UGT1419 | AAGGTAGTTAGCGGGTGTATC | GGCCAAATCGATTATACAAA |
| UGT1426 | GATGTAGGTGACTCCATCAGA | GAACTTGGTAAAGACAGCAAA |
| UGT1432 | TTCAGCTATGGAGGATATTCA | TCTTGAAGTTCTAACCGATGA |
| UGT1434 | CCTCTGACTCTGCTTATGAAA | ACATCACAATAAGTGAGCAAA |
| UGT1438 | CCTCTCTTCACGCCTTACTAT | AGTTGGATTGAGAAGTTGACA |
